# A Novel Digital Tray for Customized Professional Ozonized Gel Delivery in Periodontal Maintenance: A Case Report

**DOI:** 10.1155/crid/6467447

**Published:** 2026-10-04

**Authors:** Maurizio Pascadopoli, Matteo Pellegrini, Andrea Butera, Beatrice Romano, Andrea Scribante

**Affiliations:** ^1^ Unit of Orthodontics and Pediatric Dentistry Section of Dentistry Department of Clinical Surgical Diagnostic and Pediatric Sciences University of Pavia Pavia Italy unipv.eu; ^2^ Service of Oral Pathology Section of Dentistry Department of Clinical Surgical Diagnostic and Pediatric Sciences University of Pavia Pavia Italy unipv.eu; ^3^ Unit of Dental Hygiene Section of Dentistry Department of Clinical Surgical Diagnostic and Pediatric Sciences University of Pavia Pavia Italy unipv.eu

**Keywords:** case report, customized treatment, dentistry, nonsurgical mechanical debridement, occlusal device, ozone treatment

## Abstract

**Background:**

The use of chlorhexidine as an adjunct to nonsurgical periodontal therapy has become a standard practice. However, its use presents some limitations in terms of treatment duration and patient compliance. For these reasons, clinical research is increasingly oriented towards alternative or complementary approaches aimed at supporting mechanical debridement.

**Materials and Methods:**

This case report describes a patient affected by periodontitis who underwent nonsurgical periodontal therapy, with the application of ozone as an adjunct through Oria Bite appliance, an innovative customized tray fabricated through intraoral scanning, used for the targeted application of ozonated gel (GeliO3). Clinical periodontal parameters were evaluated at baseline, and at 1‐month and 6‐month follow‐up visits.

**Results:**

Preliminary clinical observations showed maintenance of periodontal stability, suggesting a possible role of the customized tray in supporting the control of gingival inflammation during the maintenance phase. The procedure was well‐tolerated by the patient.

**Conclusion:**

The potential role of the customized device as a complementary approach in the adjunctive treatment of periodontitis deserves further evaluation in terms of cost‐effectiveness and workflow. Future controlled clinical studies with long‐term follow‐up are required to confirm the reproducibility of the observed results.

## 1. Introduction

Periodontal disease is a chronic inflammatory condition that affects the structures supporting the teeth—including the gingiva, the periodontal ligament, and the root cementum—and is characterized by alveolar bone resorption, loss of clinical attachment, tooth mobility, and, ultimately, in advanced stages, tooth loss [1]. Periodontal disease progression may also be influenced by systemic conditions: A recent retrospective cohort study reported that, among several chronic diseases, diabetes mellitus was significantly associated with severe periodontal disease progression [2]. The cornerstone of periodontal therapy is nonsurgical periodontal treatment, such as scaling and root planing (SRP), which aims to remove supra‐ and subgingival biofilm and calculus [3]. Recent clinical studies confirm that SRP improves periodontal parameters such as probing depth and clinical attachment level in both short‐ and long‐term follow‐ups [4, 5].

Chlorhexidine (CHX) is the most widely used topical antiseptic as an adjunct to SRP due to its broad‐spectrum antimicrobial action and high substantivity; however, prolonged use has limitations, as it can cause tooth and mucosal staining, taste alterations, and changes in the oral microbiota [6, 7].

In view of these limitations, several non‐CHX–based adjunctive agents have been investigated for use in nonsurgical periodontal therapy (NSPT): A recent systematic review of randomized clinical trials reported that different natural or biologically derived formulations, including curcumin, propolis, *Morus alba*, Triphala, probiotic‐based approaches, and ozone‐based gels, may provide short‐term periodontal improvements comparable to CHX, with a potentially lower incidence of adverse effects such as tooth staining and taste alteration [8].

Ozonated gels have attracted attention as potential alternatives to CHX [9]. Randomized controlled clinical trials have reported that, when used as adjuncts to NSPT, ozone‐based gels can lead to improvements in periodontal indices comparable to those observed with CHX [9, 10]. These clinical findings may be supported by microbiological evidence showing that locally applied ozonated gel can influence microbial parameters within periodontal pockets [11]. The biological rationale of ozone‐based formulations is mainly related to their oxidative activity, which can damage microbial cell walls and cytoplasmic membranes, interfere with enzymatic systems, and increase membrane permeability; in addition, ozone has been reported to modulate inflammatory and antioxidant pathways, including Nrf2 and NF‐*κ*B signaling, thereby supporting antimicrobial activity, inflammatory response modulation, and tissue healing [12, 13].

The clinical effectiveness of locally delivered adjunctive agents may be influenced by several factors, including patient compliance, correct application, contact time, and access to periodontal sites [14]. However, these agents cannot replace mechanical biofilm control, which remains a fundamental component of periodontal therapy [3].

Digital workflows and additive manufacturing have progressively contributed to the creation of custom‐made dental devices, facilitating a better fit to the patient′s anatomical structures and ensuring greater reproducibility of clinical procedures [15]. In dentistry, these technologies have been applied to various fields [15, 16]. 3D printing has also been studied in the context of developing customized drug delivery systems [17]: The device′s geometry, porosity, and structural design appear to influence the release profile of the active ingredients [18, 19]. In periodontics, this approach could support the development of customized devices designed to improve local retention and the targeted delivery of adjuvant agents, such as ozonated gel, to selected periodontal areas [15, 19].

In this context, a custom tray was developed using intraoral scanning to enable the professional, targeted application of ozonated gel into periodontal pockets. The device was designed to improve local retention of the gel and contact with periodontal tissues, while reducing the limitations of at‐home application, such as the patient′s application skills and treatment compliance. This device is proposed as a potential adjunct in periodontal therapy, designed to support the targeted local application of ozonated gel. Therefore, the objective of this clinical case report is to describe the clinical use of a digitally customized tray for the administration of ozonated gel during periodontal maintenance therapy, focusing on feasibility, patient tolerability, short‐term periodontal outcomes after 1 month, and additional clinical follow‐up findings at 6 months.

## 2. Case Presentation

This case report describes the treatment of a 35‐year‐old man with localized periodontitis, stage I, grade A, according to the 2017 classification of periodontal and peri‐implant diseases [1]. The patient is missing his third molars as well as tooth 4.7, which had been extracted due to caries. The initial examination took place in December 2025, and the patient was enrolled in a periodontal maintenance program. The present case report was approved by the Unit Internal Review Board (IRB: 2025‐1203).

### 2.1. Diagnosis and Etiology

The 35‐year‐old patient (Figures 1 and 2) presented with a Class I occlusion on the right side, whereas on the left side there was a Class I–III molar relationship and a Class I canine relationship. The maxillary and mandibular interincisal midlines did not coincide, with a 2‐mm deviation of the mandibular midline to the left. The patient had fixed multiple‐strand retainers bonded to the palatal/lingual surfaces of the upper and lower anterior teeth. At the gingival level, the gingiva appeared pink in color, with regular contours, a firm consistency, and no obvious edema. Based on clinical examination, the patient presented with a thin periodontal phenotype. Generalized Class I recessions according to the Cairo classification [20] were present at teeth 1.3, 1.2, 2.2, 2.4, 2.5, 2.6, 2.7, 4.4, 4.3, 3.1, 3.2, 3.4, and 3.5, whereas tooth 1.6 exhibited a Cairo Class II recession associated with localized clinical attachment loss, without involvement of the furcation [20].

**Figure 1:**
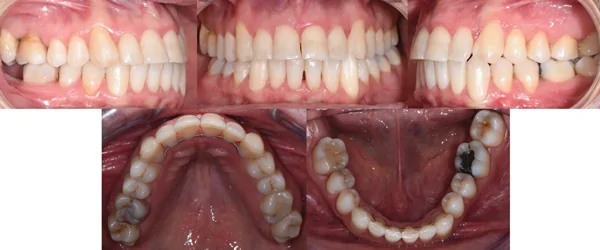
Initial intraoral photographs.

**Figure 2:**
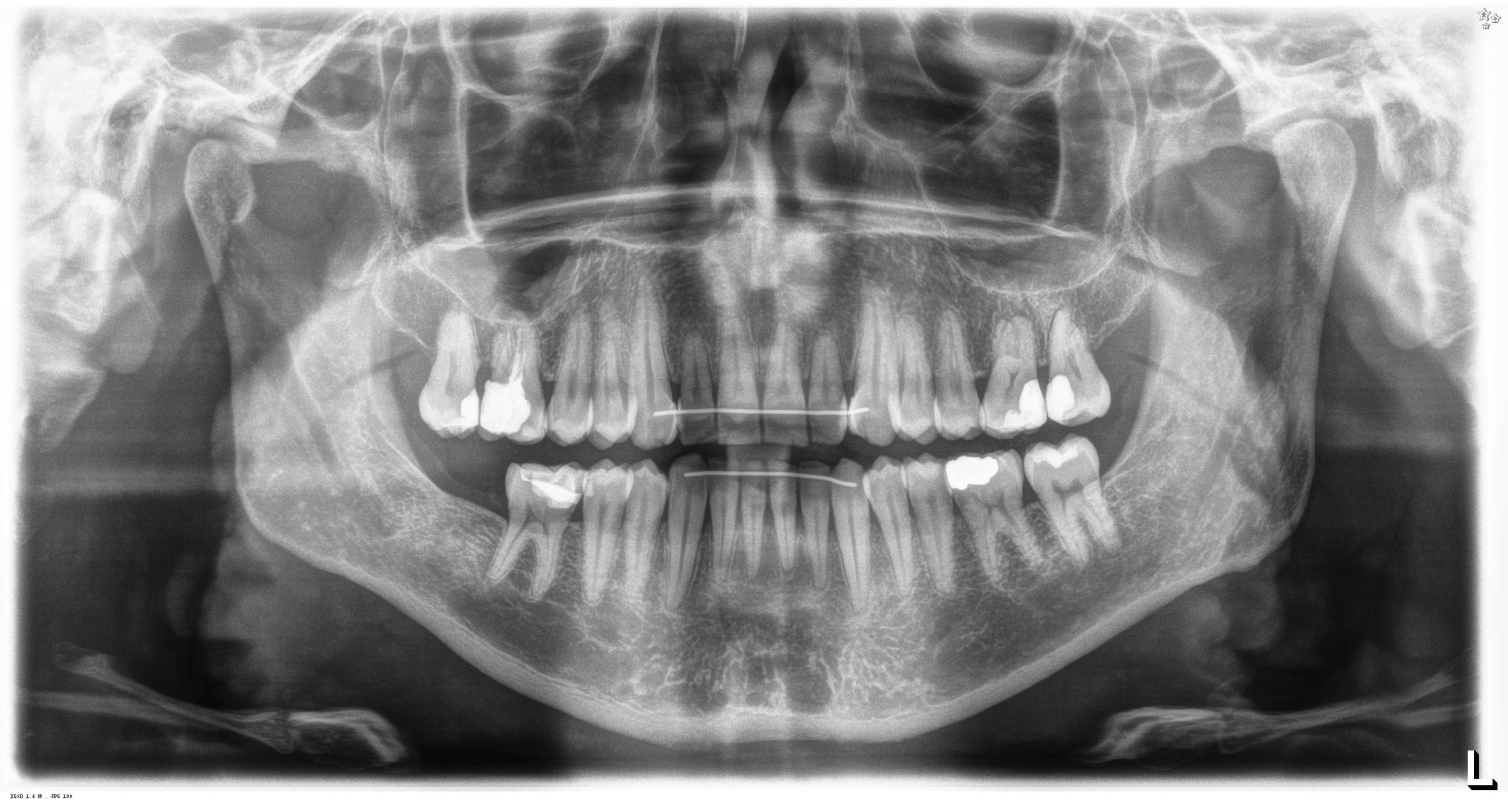
Patient′s orthopantomography dated September 2025.

The patient was a nonsmoker and had a family history of Type 2 diabetes in his maternal grandparents. The medical history was unremarkable regarding systemic diseases, allergies, and other risk factors influencing the development of periodontal disease or tissue healing. The patient was not taking any regular medications and had not taken any medications in the 15 days prior to the baseline visit.

The periodontal diagnosis was based on clinical periodontal parameters and radiographic evaluation. Periodontal involvement was localized, with the main pathological finding involving tooth 4.6, where a probing depth of 4 mm was present. Based on the recorded clinical attachment loss, probing depth, radiographic bone levels, the absence of tooth loss due to periodontal causes, and the assessment of risk factors, a diagnosis of localized periodontitis, Stage I, Grade A, was made according to the 2017 classification [1]. In this patient, plaque accumulation and insufficiently effective interdental plaque control were considered the main contributing factors, as suggested by the baseline FMPS value of 50% [21].

### 2.2. Treatment Alternatives

As previously explained, nonsurgical mechanical debridement is the gold standard for periodontal disease [3]; an initial additional option was discussed with the patient, which includes the use of topical antiseptic agents to be applied at home, such as CHX [22]. The patient was informed of the limitations of this approach, which are related to the duration of treatment, the possible occurrence of local side effects, and dependence on patient compliance, making it suitable primarily for short periods [7]. Additional non‐CHX–based options were also considered, including natural formulations and ozonated gels. After discussing the benefits and limitations of the various alternatives with the patient, it was decided to recommend the Oria Bite device (Oria Dental IT srl, Vicenza, Italy), customized via intraoral scanning and designed to deliver the ozonated gel GeliO3 (Bioemmei, Vicenza, Italy) into periodontal pockets, fissures, and enamel cavities (Figure 3).

**Figure 3:**
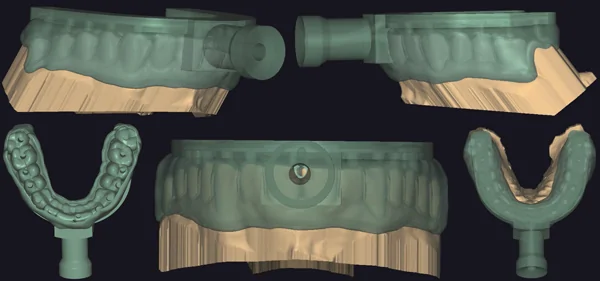
3D digital model of Oria Bite in frontal, occlusal, and lateral views, showing the conduits of the device.

After signing the informed consent form, the patient chose to undergo conventional periodontal therapy combined with the use of the occlusal device.

### 2.3. Treatment Progress

Following the preliminary evaluation, an initial appointment was scheduled to take intraoral impressions using the iTero Elements 5D intraoral scanner (Align Technology Inc., Tempe, AZ, United States), which were necessary for the fabrication of the occlusal tray (Figure 4). Subsequently, a second appointment was scheduled once the device was available. A periodontal evaluation was performed using a UNC‐15 periodontal probe (Hu‐Friedy, Chicago, IL, United States): an average periodontal pocket depth of 1.9 mm was found, with an average clinical attachment loss of 2.1 mm; a probing depth of 4 mm was present on tooth 4.6; no tooth mobility was detected; bleeding on probing was recorded at six sites per tooth and expressed as the Full‐Mouth Bleeding Score (FMBS) [23], with a score of 9.25%. Plaque accumulation was recorded on four surfaces per tooth and expressed as the Full‐Mouth Plaque Score (FMPS) [21] with a score of 50%. The patient reported using an electric toothbrush morning and evening—brushing with the right hand—and flossing in the evening; however, given the initial FMPS value of 50%, personalized instructions for at‐home oral hygiene were provided, emphasizing the correct use of the electric toothbrush and dental floss. Additional interdental aids were also introduced, including Superfloss in areas with fixed retainers and interdental brushes where indicated. NSPT was performed using a Woodpecker PT‐B piezoelectric ultrasonic device (Woodpecker Medical Instrument Co., Guilin, Guangxi, China) for supragingival and subgingival debridement. Subsequently, air polishing was performed using ABC Gentle Mini Airstream Biofilm Clearance glycine powder (Woodpecker Medical Instrument Co., Guilin, Guangxi, China); thereafter, an occlusal tray was used to apply the ozonated gel to the patient′s hard and soft tissues for 10 min (Figure 5). The gel was applied until it reached all teeth. During application of the device, the patient reported no pain, burning sensation, or mucosal irritation. Only a mild sensation of pressure was reported, with a Numerical Rating Scale score for discomfort of 3/10. A mild and transient increase in salivation was observed during device placement. At the 1‐month follow‐up, periodontal indices were recorded prior to the second application of GeliO3; subsequently, the gel was reapplied using the same custom‐made tray and re‐evaluated at 6‐month follow‐up.

**Figure 4:**
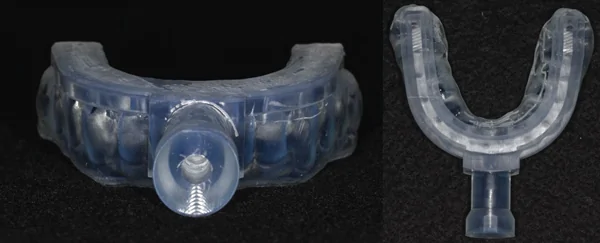
Oria Bite device.

**Figure 5:**
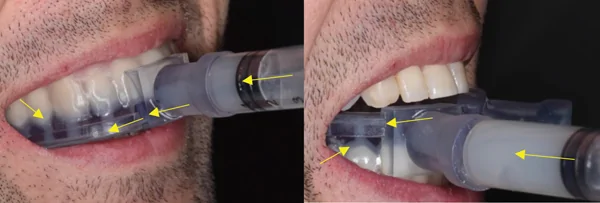
Oria Bite device during ozone administration. The yellow arrows show the flow of the ozonized gel from the syringe into the conduits that lead to dental crowns and periodontium.

### 2.4. Follow‐Up

At the 1‐month follow‐up (T1), a reduction in periodontal parameters was observed, with a mean PPD of 1.5 mm and a mean CAL of 1.7 mm. The pathological probing depth observed at tooth 4.6 returned to a physiological value of 3 mm. Furthermore, bleeding on probing was no longer detectable, with FMBS at 0%. The FMPS was 22%. No adverse effects related to the use of the ozonated gel delivery device were observed. The periodontal tissues appeared clinically healthy, with no signs of inflammation. The patient reported good tolerance of the procedure and experienced no pain, burning, or mucosal irritation during or after treatment. The patient was reevaluated during follow‐up visits and remained on periodontal maintenance therapy, with regular monitoring scheduled to assess the stability of the clinical results over time. Table 1 presents the patient′s periodontal results at baseline (T0), at the 1‐month follow‐up (T1), and at the 6‐month follow‐up (T2).

**Table 1:** Periodontal outcomes calculated at the baseline (T0), at the 1‐month follow‐up (T1), and at the 6‐month follow‐up (T2).

Periodontal outcomes	Baseline (T0)	1‐month follow‐up (T1)	6‐month follow‐up (T2)
PPD (mm)	1.9	1.5	1.6
CAL (mm)	2.1	1.7	1.6
FMBS (%)	9.25	0.00	1.00
FMPS (%)	50.00	22.00	33.00

Abbreviations: CAL, clinical attachment loss; FMBS, full‐mouth bleeding score; FMPS, full‐mouth plaque score; PPD, probing pocket depth.

## 3. Discussion

NSPT, including SRP, is the cornerstone of periodontal disease management for the treatment of periodontitis [3]; the primary goal is to remove local etiological factors above and below the gum line, the persistence of which is one of the main factors contributing to dysbiosis and gingival inflammation [24]. In certain specific cases, mechanical instrumentation alone may not be sufficient, especially in the presence of increased probing depths, root concavities, deep furrows, surface irregularities, ill‐fitting restorations, and involvement of furcation areas; these conditions can promote the persistence of biofilm, calculus, or microbial recontamination [25]. For this reason, various additional strategies have been studied with the aim of supporting biofilm control and modulating inflammation, especially in patients undergoing periodontal maintenance [3].

CHX has historically been considered one of the gold‐standard antiseptics in dentistry due to its broad‐spectrum antimicrobial activity, high retention, and ability to reduce plaque accumulation when used as an adjunct to mechanical oral hygiene procedures [22]; its widespread use is also validated in the postoperative setting, where CHX has been shown to have beneficial effects on soft tissue healing [6]. The limitations of prolonged CHX use—such as increased extrinsic staining, taste disturbances, mucosal irritation, desquamation, ulcerations, or a burning sensation [22]—and in vitro data on human gingival fibroblasts suggest that higher concentration formulations, particularly 0.2% CHX, may exert a greater cytotoxic effect, indicating the need to limit their use to selected clinical situations and for a controlled duration [7].

These limitations have fueled interest in alternative or complementary agents, including natural products, probiotics, and ozone‐based treatments, which have been studied as adjuncts to NSPT [8]. These products have garnered interest due to their antimicrobial, anti‐inflammatory, and antioxidant properties [26]. However, these products should be viewed as supportive of mechanical debridement and not as substitutes for conventional periodontal therapy, which remains based on biofilm control and supragingival and subgingival instrumentation [3].

In clinical practice, a common challenge with additional topical agents—whether of natural or synthetic origin—is the difficulty in achieving adequate local retention and sufficient contact time at subgingival sites, particularly within periodontal pockets; in fact, conventional topical formulations can be rapidly cleared from the crevicular fluid or fail to penetrate the biofilm sufficiently, thereby reducing their therapeutic efficacy [11]. When these products are applied at home, the clinical outcome can be influenced by patient adherence, proper execution of the protocol, and maintenance of adequate oral hygiene practices—all of which are central to supportive periodontal care [3]. This challenge underscores the interest in more targeted professional delivery systems capable of promoting local retention and contact between the active agent and periodontal tissues.

In light of the limitations associated with the prolonged use of CHX and the challenges common to at‐home topical agents, this case report evaluated the professional application of an ozonated gel (GeliO3) using a custom‐made occlusal device (Oria Bite). Ozone was chosen for its antimicrobial, anti‐inflammatory, and immunomodulatory properties: Its bactericidal effect is primarily linked to oxidative mechanisms, involving alterations in cell walls and microbial membranes, increased permeability, and interference with essential enzymatic systems [12]. Furthermore, ozone can modulate inflammatory and oxidative stress pathways, including the Nrf2 and NF‐*κ*B pathways, thereby influencing cytokine production and the antioxidant systems involved in the tissue response and healing processes [12, 13]. The use of the custom‐made Oria Bite tray has been proposed with the aim of facilitating the professional application of the gel and potentially improving its local retention and contact with periodontal tissues, allowing the practitioner to control the procedure and variables such as the amount of product used, proper distribution, and adherence to contact time, while remaining a supplement to conventional mechanical therapy and not a substitute for it. The device is manufactured using a digital workflow to achieve a customized fit to the patient′s dental anatomy. From a clinical standpoint, professional application using a custom tray can reduce some of the limitations of additional at‐home therapies, but it still requires patient cooperation: The patient must attend scheduled appointments, tolerate the device for 10 min, maintain proper oral hygiene, and adhere to the periodontal maintenance program. The term “standardization” must be interpreted with caution, since in this case the gel delivery pressure was not measured instrumentally and, therefore, may still depend on the operator: The device can be considered a tool designed to facilitate a more targeted and repeatable application, but it does not allow for definitive conclusions regarding the actual standardization of administration.

From a practical standpoint, it is important to consider that, compared to a simple at‐home topical application of the gel, the use of a custom tray involves a more complex clinical and technical workflow. This approach requires digital data acquisition, CAD/CAM design, manufacturing or laboratory procedures, specialized materials, lead times, and at least one additional appointment prior to clinical application. In the literature, digital dental workflows have been associated with potential organizational and precision benefits, but also with higher laboratory and equipment costs compared to conventional approaches, particularly regarding materials, software, machinery, and production stages [16]. On the other hand, these additional costs and time requirements could be justified if future studies demonstrate a real advantage in terms of gel retention, procedural reproducibility, tolerability, clinical outcomes, or reduced reliance on at‐home application. Therefore, at this time, the cost‐effectiveness of the custom tray compared to simpler conventional methods of local gel application remains to be determined.

Regarding the clinical data observed in the patient, the improvements noted cannot be attributed specifically to the ozonated gel or the custom tray, since SRP, air polishing with glycine, reinforcement of oral hygiene instructions, and application of the ozonated gel were all performed during the same treatment session: The reduction in FMPS from 50% to 22% suggests that improved home plaque control may have contributed to the reduction in bleeding and inflammatory parameters; the device was found to be easy to use and well tolerated by the patient, with no pain, burning, or mucosal irritation, and only a mild sensation of pressure. Therefore, the device offers plausible practical advantages and, in the case described, proved feasible and well‐tolerated; however, its efficacy or superiority compared to other methods of local application remains to be determined in future studies.

This case report has inherent limitations. The observations are based on a single patient, and the absence of a control site, a comparison group, or a follow‐up period after mechanical debridement alone makes it impossible to distinguish the specific contribution of the ozonated gel and the custom‐made tray from the effects of SRP, glycine air polishing, and improved home oral hygiene. Periodontal measurements at follow‐up were recorded before the second application of the ozonated gel; therefore, the 1‐month data represent the short‐term outcome of the initial protocol, whereas the 6‐month data represent a more extended clinical observation of the second gel application, but remain limited to a single clinical case. A further limitation concerns the absence of an instrumental measurement of delivery pressure during gel administration; consequently, the degree of standardization of the procedure remains to be verified in controlled studies.

Future controlled clinical studies, using a split‐mouth or parallel‐group design, are needed to evaluate the long‐term effects of the professional application of ozonated gel using a custom‐made tray and to distinguish the contribution of the device and the ozonated gel from that of mechanical debridement. Further studies should include patient‐reported outcomes, microbiological analyses, assessment of delivery pressure, clinical timelines, and cost‐effectiveness analysis, in order to better define the clinical indications for this approach.

## 4. Conclusions

The professional application of ozonated gel using the Oria Bite could be a promising approach in adjuvant nonsurgical periodontal treatment. The patient‐specific design of the device may support a more targeted and repeatable application of the gel, and potentially improve its local retention and contact with periodontal tissues during periodontal maintenance therapy. However, given the fact that this is a case report without a control site or comparison group, and because nonsurgical periodontal debridement, glycine air polishing, oral hygiene reinforcement, and ozonated gel application were performed within the same treatment protocol, the observed clinical improvements cannot be attributed specifically to the gel or to the customized tray. The 1‐month and 6‐month follow‐up findings support the clinical feasibility and tolerability of this adjunctive approach, but further controlled studies are needed to assess its efficacy, long‐term stability, cost‐effectiveness, and potential advantages over simpler methods of local gel application.

## Author Contributions

Conceptualization, Maurizio Pascadopoli and Andrea Scribante; methodology, Maurizio Pascadopoli and Andrea Scribante; investigation, Beatrice Romano; resources, Andrea Butera; data curation, Beatrice Romano and Maurizio Pascadopoli; writing—original draft preparation, Beatrice Romano and Maurizio Pascadopoli; writing—review and editing, Andrea Scribante; visualization, Beatrice Romano and Matteo Pellegrini; supervision, Andrea Butera and Andrea Scribante; project administration, Matteo Pellegrini.

## Funding

Open access publishing facilitated by Universita di Pavia, as part of the Wiley ‐ CRUI‐CARE agreement.

## Disclosure

All authors have read and approved the final version of the manuscript. Andrea Scribante had full access to all of the data in this study and takes complete responsibility for the integrity of the data and the accuracy of the data analysis. Oria Bite device is subject to a pending Italian (IT 102024000022626) and International Patent request (WO 2026/078071 A1).

## Ethics Statement

The study was conducted in accordance with the Declaration of Helsinki and approved by the Unit Internal Review Board (IRB: 2025‐1203, date of approval: December 3, 2025).

## Consent

Written informed consent was obtained from the patient included in the study.

## Conflicts of Interest

Prof. Andrea Scribante is the co‐holder of Oria Bite patent.

## Supporting information


**Supporting Information** Additional supporting information can be found online in the Supporting Information section.

## Data Availability

The authors confirm that the data supporting the findings of this study are available within the article.
