# Supplementary material for: A Novel Digital Tray for Customized Professional Ozonized Gel Delivery in Periodontal Maintenance: A Case Report
Source: Case Rep Dent. 2026 Oct 4;2026:6467447. doi: 10.1155/crid/6467447 (PMC13635482; doi:10.1155/crid/6467447)
Supplement: Supplementary file 1 [file CRID-2026-6467447-s001.pdf]

CARE
case report guidelines

CARE Checklist of information to include when writing a case report

(cc) BY-NC-ND

☑

Topic	Item	Checklist item description	Reported on Line
Title	1	The diagnosis or intervention of primary focus followed by the words "case report"	2–3
Key Words	2	2 to 5 key words that identify diagnoses or interventions in this case report, including "case report"	33–34
Abstract (no references)	3a	Introduction: What is unique about this case and what does it add to the scientific literature?	20–23
	3b	Main symptoms and/or important clinical findings	26–29
	3c	The main diagnoses, therapeutic interventions, and outcomes	23–29
	3d	Conclusion—What is the main "take-away" lesson(s) from this case?	29–32
Introduction	4	One or two paragraphs summarizing why this case is unique (**may include references**)	71–78
Patient Information	5a	De-identified patient specific information.	80–84
	5b	Primary concerns and symptoms of the patient.	not applicable
	5c	Medical, family, and psycho-social history including relevant genetic information	95–98
	5d	Relevant past interventions with outcomes	81–82; 89
Clinical Findings	6	Describe significant physical examination (PE) and important clinical findings.	86–105
Timeline	7	Historical and current information from this episode of care organized as a timeline	135–181
Diagnostic Assessment	8a	Diagnostic testing (such as PE, laboratory testing, imaging, surveys).	99–105; 138–142
	8b	Diagnostic challenges (such as access to testing, financial, or cultural)	not applicable
	8c	Diagnosis (including other diagnoses considered)	80–84; 99–103
	8d	Prognosis (such as staging in oncology) where applicable	80; 102
Therapeutic Intervention	9a	Types of therapeutic intervention (such as pharmacologic, surgical, preventive, self-care)	119–127
	9b	Administration of therapeutic intervention (such as dosage, strength, duration)	147–156
	9c	Changes in therapeutic intervention (with rationale)	not applicable
Follow-up and Outcomes	10a	Clinician and patient-assessed outcomes (if available)	173–189
	10b	Important follow-up diagnostic and other test results	173–189
	10c	Intervention adherence and tolerability (How was this assessed?)	150–154; 177–178
	10d	Adverse and unanticipated events	176
Discussion	11a	A scientific discussion of the strengths AND limitations associated with this case report	254–265
	11b	Discussion of the relevant medical literature **with references**.	192–227
	11c	The scientific rationale for any conclusions (including assessment of possible causes)	250–257
	11d	The primary "take-away" lessons of this case report (without references) in a one paragraph conclusion	272–280
Patient Perspective	12	The patient should share their perspective in one to two paragraphs on the treatment(s) they received	not applicable
Informed Consent	13	Did the patient give informed consent? Please provide if requested	Yes ☒ No ☐
